# PMA2020: Rapid Turn‐Around Survey Data to Monitor Family Planning Service and Practice in Ten Countries

**DOI:** 10.1111/sifp.12031

**Published:** 2017-08-28

**Authors:** Linnea Zimmerman, Hannah Olson, PMA2020 Principal Investigators Group, Amy Tsui, Scott Radloff

## CONTENTS

### Research Questions for Original Data Collection

In 2012, the London Summit on Family Planning adopted the ambitious goal of increasing access to contraception for 120 million additional women and girls in the world's poorest countries by 2020. Family Planning 2020 (FP2020)^1^ was established as a coordinating body to monitor progress. In order to monitor country progress and to change course in the event of stagnating or declining use, data were needed more frequently and more quickly than data provided by typical surveys. Performance Monitoring and Accountability 2020 (PMA2020) was created to provide rapid and frequent estimates of modern contraceptive use in FP2020 priority countries. Currently operational in ten countries (Burkina Faso, DRC, Ethiopia, Ghana, India, Indonesia, Kenya, Niger, Nigeria, and Uganda), PMA2020 conducts surveys every six months to one year, providing FP2020, governments, and other stakeholders frequent information on contraceptive use, demand, and supply that can inform policies and programs and identify areas for improvement.

PMA2020 recruits women from within or near selected enumerations areas (EAs) and trains them in collecting household and facility‐level data using smartphones and submitting the data to a cloud server. These resident enumerators (REs) are then deployed to collect data on a repeated basis—each round within a six‐week period—with refresher training between each round.

Household data include information on household members, as well as assets, livestock ownership, housing construction, and water, sanitation, and hygiene (WASH) conditions. Women aged 15–49 who are either usual members of the household or who slept in the household the night before the interview are eligible for the female interview. The female survey gathers information on sociodemographic characteristics, such as education and marital status, as well as measures of fertility and contraceptive use, including the dates of women's first and two most recent births, age at first sex, age at first marriage, and age and parity at first contraceptive use. Family planning measures include current use of contraception and contraceptive use within 12 months preceding the interview among current non‐users, by method previously used. The data also include reasons for not using or stopping a method of contraception, intention to use contraception in the future among non‐users, autonomy and influences related to contraceptive decision‐making, and the “method information index”—whether she was told about any methods other than the one she chose, whether she received counseling on side effects, and whether she was told what to do if she experienced side effects. Several additional quality and choice indicators can be calculated. Constructed variables in the dataset include wealth quintiles/tertiles, unmet need for family planning, and current use of a modern contraceptive method.

While family planning is the focus of the survey, a small number of water and sanitation questions have been added to the household, female, and service delivery point (SDP) questionnaires. The WASH questions that are asked in the household and female surveys are round‐specific and cover topics such as distance to a water source, handling of child waste, diarrhea prevalence among children under 5 years of age, and menstrual hygiene management. The range of topics covered to date demonstrates the flexibility of the PMA platform: once the data collection platform is established, data can be collected to support other areas of health intervention. Such expansions are underway in selected countries with respect to primary health care, maternal and newborn health, schistosomiasis, and nutrition.

Data for SDPs are collected using a health facility questionnaire that is fielded concurrently with the household/female data collection. The SDP dataset includes measures of contraceptive availability, stock‐outs, numbers of clients served, outreach through mobile services and community health workers, and integration of family planning with other health services such as HIV, maternal health, and post‐abortion care. The SDP dataset also includes measures of service quality, such as availability of supplies for both insertion and removal of intrauterine devices (IUDs) and implants, storage conditions for contraceptive commodities, and availability of adequate hand‐washing stations for providers. The SDP dataset includes variables that identify the enumeration areas that each SDP serves. These enumeration areas are the same EAs as in the household dataset, which allows for linking at the community level between households and the health service environment.

### Sample Selection and Size, including Response Rates and Loss to Follow‐Up

The PMA2020 household and female survey uses a multi‐stage cluster sample design to draw a probability sample of households and eligible females. The indicator used to calculate the female survey sample size is the modern contraceptive prevalence rate (mCPR) among all women aged 15–49 with a maximum margin of error of ±3 percentage points at the national level and a maximum of ±5 percentage points for urban/rural strata. In some countries sample sizes are sufficient to produce sub‐national estimates, generally at the next lowest administrative level. Country‐specific sampling descriptions specify the level at which estimates are representative. Table 1 summarizes the administrative levels at which the estimates are representative and the associated margins of error.

**Table 1:** Level at which data are representative and margin of error by country

Country	Level at which data are representative	Margin of error used for original sample size calculation
Ghana	National	<2%
	Urban/Rural	<3%
DRC	Kinshasa	<2%
	Kongo Central	<2%
Ethiopia	National	<2%
	Urban/Rural	<3%
	5 regions^a^	5%
Uganda	National	2%
	Urban/Rural	<3%
Kenya	National	<3%
	Urban/Rural	3%
	9 counties^b^	5%
Burkina Faso	National	2%
	Urban/Rural	<5%
Nigeria	National	<2%
	Urban/Rural	<2%
	7 states^c^	<2‐3%
Niger	National	<2%
	Niamey	3%
	Urban/Rural	<3%
Indonesia	National	<2%
	Urban/Rural	<3%
	South Sulawesi	<3%
	Makassar district	5%
India	Rajasthan	2%
	Urban/Rural	3%

a Addis, Amhara, Oromiya, Tigray, and SNNPR

b Bungoma, Kericho, Kiambu, Kilifi, Kitui, Nairobi, Nandi, Nyamira, Siaya

c Anambra, Kaduna, Kano, Lagos, Nasarawa, Rivers, Taraba

Each EA has approximately 200 households. At the EA level, the RE lists and maps all households and private health facilities. She is assigned a random selection of 33–44 households (depending on the country) and obtains the consent of household and female respondents for interviews. All data collection is approved by country‐specific IRBs.

Private SDPs are included in the sample if they fall within the boundaries of the enumeration areas. Up to three randomly chosen private facilities are selected from each EA. Public health facilities are included in the sample if the selected EA falls into the catchment area of the SDP. Implementing partners obtain a list of public health facilities assigned to provide services to residents in the selected EAs; facilities at the lowest level (equivalent to a health post), secondary level (e.g., health center), and tertiary level (e.g., referral hospital) are selected into the sample. The SDP sample thus reflects the services available to a representative population, rather than being representative of all SDPs in the country. If a national frame of SDPs, both public and private, is available, the PMA SDP sample can be weighted to provide measures representative of facilities at the national level.

Subsequent survey rounds are conducted in the same EAs, but with a new sample selection of households. SDPs tend to be the same facilities between survey rounds since the public‐sector facilities that serve a particular enumeration area are not likely to change. If there are more than three private facilities within an enumeration area, three are randomly selected in each round. At the fifth round of data collection, new enumeration areas are selected to limit respondent fatigue and possible interview effects in the community. New enumeration areas are generally geographically contiguous to the original EAs and share the same urban/rural designation.

Table 2 shows the response rates for each survey and round by household and the complete female sample (both usual household members and visitors). Analyses conducted by PMA2020 include only regular household members. Table 3 shows the rates for the SDP data. Since PMA2020 data are cross‐sectional, there is no follow‐up and thus no loss to follow‐up.

**Table 2:** Household and Female Response Rates Across PMA2020 Surveys

Country and round	Data collection period	Households selected	Households occupied	Households interviewed	Household response rate (%)	Total eligible women	Eligible women interviewed	Eligible women response rate (%)
Ghana Round 1	Oct‐Dec 2013	4072	3910	3536	90.4	4191	3708	88.9
Ghana Round 2	Jan‐May 2014	4148	3802	3419	89.9	4264	3974	93.5
Ghana Round 3	Oct‐Dec 2014	4164	4072	3927	96.4	4806	4621	96.6
Ghana Round 4	May‐Jun 2015	4186	4142	4053	97.9	5391	5234	97.5
Ghana Round 5	Aug‐Dec 2016	4182	4118	4062	98.6	3860	3746	97.0
DRC/Kinshasa Round 1	Nov 2013‐Jan 2014	*	*	1777	*	2160	2132	98.7
DRC/Kinshasa Round 2	Aug‐Sep 2014	*	*	1900	*	3017	2877	95.4
DRC/Kinshasa Round 3	May‐Jun 2015	1844	1828	1768	96.7	2841	2683	95.3
DRC/Kinshasa Round 4	Nov‐Dec 2015	1918	1843	1774	96.3	2869	2741	96.1
DRC/Kongo Central Round 4	Nov‐Dec 2015	1720	1688	1625	96.3	1653	1573	95.4
DRC/Kinshasa Round 5	Aug‐Sep 2016	1914	1895	1841	97.2	2733	2593	94.9
DRC/Kongo Central Round 5	Aug‐Sep 2016	1715	1641	1575	96.0	1756	1688	96.1
Ethiopia Round 1	Jan‐Mar 2014	6979	6919	6772	97.9	6688	6514	97.6
Ethiopia Round 2	Sep‐Nov 2014	6997	6927	6813	98.4	6888	6713	97.5
Ethiopia Round 3	Apr‐Jun 2015	7735	7703	7643	99.2	7708	7628	99.0
Ethiopia Round 4	Mar‐May 2016	7732	7695	7651	99.4	7642	7537	98.6
Uganda Round 1	Apr‐Jun 2014	4802	4576	4257	93.0	3987	3754	94.4
Uganda Round 2	Jan‐Feb 2015	4840	4429	4143	93.5	3859	3654	94.7
Uganda Round 3	Aug‐Oct 2015	4838	4671	4412	94.5	3889	3705	95.3
Uganda Round 4	Mar‐Apr 2016	4839	4433	4191	94.5	4044	3816	94.4
Kenya Round 1	May‐Jul 2014	5040	4859	4518	93.0	3987	3792	95.7
Kenya Round 2	Nov‐Dec 2014	5038	4803	4604	95.9	4470	4370	97.9
Kenya Round 3	May‐Jun 2015	5040	4958	4810	97.0	4506	4433	98.4
Kenya Round 4	Nov‐Dec 2015	5039	4928	4792	97.2	5025	4960	98.7
Kenya Round 5	Nov 2016–Jan 2017	6343	6239	6073	97.3	6050	5975	98.8
Burkina Faso Round 1	Nov 2015‐Jan 2016	1857	1810	1760	97.2	2220	2094	94.3
Burkina Faso Round 2	Apr‐Jun 2015	1855	1778	1733	97.5	2270	2150	94.7
Burkina Faso Round 3	Mar‐Apr 2016	2906	2864	2803	97.9	3493	3353	96.1
Burkina Faso Round 4	Nov 2016‐Jan 2017	2904	2807	2751	98.0	3414	3245	95.1
Nigeria/Kaduna Round 1	Sep‐Oct 2014	2309	2287	2194	95.9	2637	2575	97.9
Nigeria/Lagos Round 1	Sep‐Oct 2014	1302	1233	974	79.0	864	771	89.3
Nigeria/Kaduna Round 2	Sep‐Oct 2015	2308	2288	2264	99.0	3006	2943	97.9
Nigeria/Lagos Round 2	Sep‐Oct 2015	2080	1982	1777	89.7	1617	1449	89.8
Nigeria National Round 3	May‐Jul 2016	10815	10436	10131	97.1	11463	11150	97.4
Niger/Niamey Round 1	Jul‐Aug 2015	1155	1143	1129	98.8	1408	1352	96.0
Niger National Round 2	Feb‐Apr 2016	2894	2833	2787	98.4	3193	3042	95.3
Niger/Niamey Round 3	Nov‐Dec 2016	1146	1127	1099	97.5	1443	1410	97.7
Indonesia Round 1	Jun‐Aug 2015	12963	12537	11726	93.5	11618	10566	91.0
India/Rajasthan Round 1	May‐Sep 2016	5116	5002	4870	97.4	5741	5456	95.0

^*^In DRC Rounds 1 and 2, only household forms that were completed were uploaded and saved. It is thus not possible to calculate % of households occupied or non‐response rates for these two rounds.

**Table 3:** SDP Response Rates Across PMA2020 Surveys

Country and round	SDPs identified	SDPs completed	SDP response rate (%)
Ghana Round 1	143	138	96.5
Ghana Round 2	132	124	93.9
Ghana Round 3	241	231	95.9
Ghana Round 4	239	233	97.5
Ghana Round 5	176	157	89.2
DRC/Kinshasa Round 2*	257	248	96.5
DRC/Kinshasa Round 3	255	248	97.3
DRC/Kinshasa Round 4	239	228	95.4
DRC/Kongo Central Round 4	122	120	98.4
DRC/Kinshasa Round 5	185	173	93.5
DRC/Kongo Central Round 5	105	102	97.1
Ethiopia Round 1	397	389	98.0
Ethiopia Round 2	407	398	97.8
Ethiopia Round 3	453	445	98.2
Ethiopia Round 4	461	456	98.9
Uganda Round 2*	373	362	97.1
Uganda Round 3	379	363	95.8
Uganda Round 4	384	350	91.1
Kenya Round 1	277	264	95.3
Kenya Round 2	354	324	91.5
Kenya Round 3	359	348	96.9
Kenya Round 4	358	338	94.4
Kenya Round 5	429	410	95.6
Burkina Faso Round 1	107	106	99.1
Burkina Faso Round 2	107	100	93.5
Burkina Faso Round 3	134	132	98.5
Burkina Faso Round 4	135	131	97.0
Nigeria/Kaduna Round 1	137	135	98.5
Nigeria/Lagos Round 1	94	87	92.6
Nigeria/Kaduna Round 2	152	148	97.4
Nigeria/Lagos Round 2	132	123	93.2
Nigeria National Round 3	694	667	96.1
Niger/Niamey Round 1	33	31	93.9
Niger National Round 2	138	132	95.7
Niger/Niamey Round 3	30	27	90.0
Indonesia Round 1	940	885	94.1
India/Rajasthan Round 1	308	294	95.6

^*^No Round 1 SDP survey was conducted in this country/round

### Data Quality

PMA2020 employs automated checks to monitor and improve data quality. Progress and error reports are run daily by in‐country data managers. These reports track progress in the number of interviews conducted and transmitted to the server, monitor response rates, and identify potential data quality issues, including flagging questions and interviewers with high rates of non‐response, flagging missing or incomplete forms, and using GPS locations to track the geographic distribution of interviews. Additionally, PMA2020 has developed tools called “PMA Analytics” that record how long each question appears on the screen before moving forward. This is a proxy for the amount of time it takes to ask and record each response, which is useful for identifying any falsified or questionable data.

**Figure 1:**
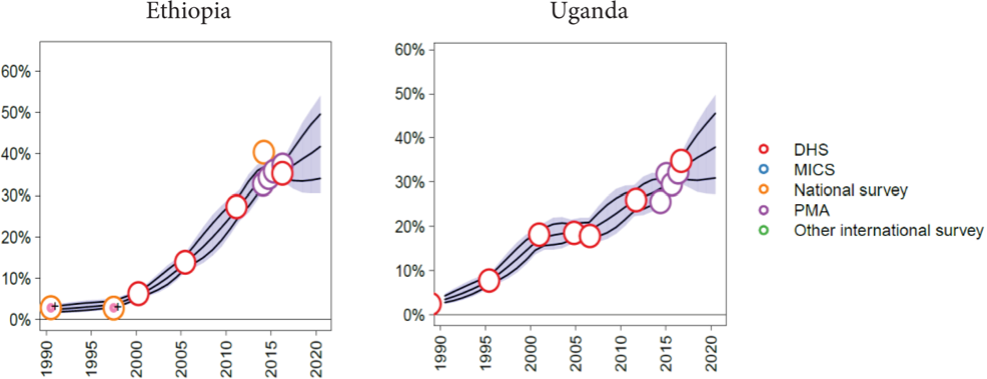
Trends in modern contraceptive prevalence rate among married women in Ethiopia and Uganda NOTE: Generated using FPET tool on April 10, 2017. Citation: New, JR and Alkema, L (2015). Family Planning Estimation Tool (FPET). Available at http://fpet.track20.org/

Estimates of modern contraceptive use, the key indicator used to determine sample size, have been broadly consistent across countries and rounds. Figure 1 shows mCPR estimates for married women in Ethiopia and Uganda generated by the FPET models used by Track20.^2^ The PMA estimates are consistent with trends shown by other estimates and indicate increases across rounds, with some variability. There is variability over time in all countries due to sampling error, but overall the estimates for mCPR show consistent increases. New EAs are selected in Round 5 in the event that family planning awareness at the community level has increased as a result of RE interviews. This addition allows further consistency comparisons.

Despite robust data checks, reporting biases and measurement errors may occur. To provide additional information on its design and protocols, PMA is introducing a series of methodological reports, available through the website, that summarize and review data quality issues and the effect these may have on estimates. The first such report, “Response patterns on behavioral outcomes in relation to use of resident enumerators over multiple survey rounds,” reviews the effect of using resident enumerators on response patterns. Future reports will explore such topics as the effect of date misreporting and results from PMA Analytics.

### Data Formats

PMA2020 data are available in a variety of formats, including pre‐calculated indicators, interactive tables, and individual and household‐level microdata. Pre‐calculated indicators presented in Snapshot of Indicators (SOI) tables, charts (DataLab), and published briefs are available on the PMA2020 website (www.pma2020.org). Estimates for both DataLab and SOI tables are generated using standard Stata do‐files and cross‐checked between DataLab and SOI tables for consistency prior to being published.

After each round of data collection, priority FP indicators and figures are made available in the “Key Family Planning Indicator Briefs,” and detailed analyses of priority indicators disaggregated by standard demographic characteristics are available through the SOI tables and PMA2020 DataLab. Descriptions of the original sample selection and any round‐specific updates to the sampling, round‐specific questionnaires, response rates, and sample error estimates are published on the website, accompanying each SOI table. The PMA website also contains additional memos describing the sampling procedure and assumptions used by PMA, general guidance on the construction of sample weights, and description and definition of key indicators. Sample errors are provided for key indicators in additional tables.

Microdata are available in csv, xls, or dta format. While PMA2020 data are cleaned during data collection, very little is done to change the content of the data; that is, missing values are not imputed, and extreme values are not corrected. Content is changed only when a skip pattern necessitates a correction in the data, such as making the date of first birth and the date of most recent birth the same value for women who have had only one child. Otherwise, non‐response and extreme values are left to be corrected at the discretion of the analyst. All observations are provided, including interviews that were not completed, to allow users to reconstruct response rates. All identifying information, including names and sub‐regional geographic identifiers, are deleted prior to release to protect the anonymity of PMA2020 respondents.

### How and When Data Were Collected

Since its inception in April 2013, the PMA2020 project, in partnership with country research organizations, has completed 39 nationally or sub‐nationally representative household and health facility surveys in ten low‐ or middle‐income countries. Dates of data collection are provided in Table 2.

All interviews with household, female, and SDP respondents are conducted face‐to‐face, and responses are entered into an Android smartphone using Open Data Kit (ODK) software. Following the interview, data are submitted to a secure cloud server, where they are instantly aggregated. Data are monitored daily by in‐country data management and quality assurance teams, with technical assistance provided by the PMA2020 team at Johns Hopkins University (JHU). Fieldwork is generally completed within 4–6 weeks, with preliminary cross‐tabulations of the data prepared as charts and tables within another 4–6 weeks.

The majority of questions included in the PMA2020 household and female questionnaires replicate wording from the Demographic and Health Surveys (DHS), and many of those used in the SDP survey replicate questions in the Service Provision Assessment (SPA). In terms of measurement reliability, most results from the two types of surveys should be directly comparable. The PMA2020 female and SDP survey questionnaires are designed to measure indicators that are essential to FP2020 and national family planning efforts. This constraint on content keeps the questionnaire focused and brief enough to be administered in a short period.

### Data Location and Access

PMA2020 microdata are accessible on request through the project website (http://www.pma2020.org/request-access-to-datasets-new) upon approval by PMA2020's coordinating center at JHU in Baltimore. Datasets are made publicly available approximately six months after data collection is completed. To view publicly available datasets and to obtain online access to PMA2020 datasets, users must create an account and submit a brief description of the research question. Requests can be submitted in either English or French. While the dataset language (variables/value labels) is English, dataset user notes and the codebook are also available in French for DRC, Niger, and Burkina Faso.

Users granted access will be linked to a website from which the relevant materials can be downloaded. If a dataset is updated, users who have received approval to download the data will be notified by email. Only one version of each dataset will be available through the website. All datasets are archived and specific datasets can be made available upon request and review.

## USE

Estimates in the two‐page family planning briefs are preliminary based on having a minimum of 95 percent of expected interviews submitted; there may be slight variation in those estimates and estimates presented in the DataLab or SOI tables. Estimates provided in the DataLab and SOI tables are based on final datasets that are released publicly and should be consistent between the two sources. The SOI tables will indicate whether estimates are based on small sample sizes; DataLab does not do so. Particularly for SDP indicators, it is recommended that users consult both sources to check adequacy of sample sizes.

Although user support for analysis of the microdata is limited, the dataset user notes provide a brief description of the variables that can help identify households, individual females, and SDPs. The notes detail country‐specific variables and other variables of interest, including constructed ones generated for analysis. The notes also include a brief sample description, details on the criteria PMA2020 employs for inclusion in the analytic sample, and explanations of any anomalies in the data. If a dataset has been updated, the user notes will list the variables that were changed and indicate the changes made. It is recommended that users review the dataset notes before analyzing the data. Given the constraints in using ODK software, both the month and year of a date must be entered. If a date is entirely unknown, it is entered as January 1, 2020. If the year is known but the month is not, the default month is set to January. It is recommended that analysts review the distribution of events by month.

In‐country partner institutions reserve the right to limit access to selected variables for up to one year if the data collection is funded through partner relationships that require restricted access.

It is suggested that publications based on PMA2020 data include the following citation:

Performance Monitoring and Accountability 2020 (PMA2020) Project, [name of the relevant PMA2020 partner institution(s)]. [Survey year]. [Country]. Baltimore, MD: PMA2020, Bill & Melinda Gates Institute for Population and Reproductive Health, Johns Hopkins Bloomberg School of Public Health.

The suggested citation is provided with the datasets.

## VALUE OF THE DATA

The unique scientific value of PMA2020 survey data lies in the following features:

- PMA2020 provides nationally representative survey data on family planning indicators with rapid turnaround on an annual or more frequent basis.
- It collects information directly from facilities that provide family planning services to the sampled households. By combining both facility and household components of the PMA2020 platform, researchers can set up both the supply and demand sides for analysis of the association between family planning service delivery outputs and the population outcomes in a way that few other facility surveys can. This allows for the generation of unique insights and hypothesis‐testing.
- The selected geographic locations for PMA2020 surveys prioritize the FP2020 pledging countries to serve as a monitoring platform for ensuring that FP2020 goals and commitments are being met.
- In addition to providing comparable measures of core FP indicators, PMA2020 gathers information on emerging issues in FP and reproductive health that other large‐scale surveys do not capture. PMA has included questions in selected countries on implant removal, menstrual hygiene management, Sayana Press introduction, emergency contraceptive use, abortion, contraceptive acceptability, and program exposure.
- Enumeration areas and resident enumerators that are included in multiple rounds are masked with the same identifiers across rounds. It is thus possible to link interviews conducted in the same geographic area or conducted by the same interviewer over time, allowing for the investigation of longitudinal change and/or interviewer effects over time.

## Footnotes

1 See http://www.familyplanning2020.org.

2 The Family Planning Estimation Tool (FPET) is a web application that provides country‐specific estimates of contraceptive prevalence and unmet need for family planning. The global implementation of the estimation approach is described in L. Alkema et al., “National, regional and global rates and trends in contraceptive prevalence and unmet need for family planning between 1990 and 2015: A systematic and comprehensive analysis,” *The Lancet*, 2013, 381(9878):1642–1652. Available at http://www.thelancet.com/journals/lancet/article/PIIS0140-6736(12)62204-1/abstract.
